# Comparative Effectiveness of Olanzapine Versus Haloperidol in Treating Delirium: A Systematic Literature Review and Meta-Analysis of Randomized Trials

**DOI:** 10.1192/j.eurpsy.2025.1544

**Published:** 2025-08-26

**Authors:** D. Soler Lopes, I. B. De Oliveira, M. L. Geremias, A. L. Stephany

**Affiliations:** 1 Lancaster University, Lancaster, United Kingdom; 2 USP, Sao Paulo; 3 UNIVILLE, Joinvile; 4 Serviço ntegrado de Saúde, Jacareí, Brazil

## Abstract

**Introduction:**

Delirium, as described in the DSM-V, is a disruption in attention and consciousness that develops over a brief period, representing an acute change from baseline awareness. First-generation antipsychotics, such as haloperidol, are often advised as the first line of pharmacological treatment. In comparison to haloperidol, olanzapine appears to be more beneficial in terms of efficacy and safety, according to a 2016 systematic review and meta-analysis of randomized clinical studies. However, most of the research included were single-center investigations with tiny sample numbers, diverse study demographics, and bias potential.

**Objectives:**

The aim of this systematic review was to identify the current best evidence on the effectiveness of olanzapine versus haloperidol in various clinical settings to guide best practices for healthcare professionals. Also, this literature review seeks to provide a up-to-date synthesis of the current evidence on this subject.

**Methods:**

We conducted a systematic search of four databases (PubMed, PsycINFO, CINAHL, and Cochrane Central) from inception through January 31st, 2024, using keywords related to delirium (acute confusion, confusion state, confusional state), olanzapine, and haloperidol. The search was limited to randomized controlled trials comparing olanzapine with haloperidol, without restrictions on dose, route of administration, or drug exposures. When analyzing outcomes with a robust number of studies, we applied a random-effects model. For outcomes with fewer studies, we used a fixed-effects model. Additionally, we conducted sensivity and subgroup analyses. All statistical evaluations were performed using the RevMan software.

**Results:**

Seven studies met our inclusion criteria. Haloperidol was associated with a significantly lower severity of delirium after 2-3 days of treatment compared to olanzapine, with a small effect size (g = 0.40, 95% CI [0.02; 0.78], p = 0.04) based on three studies (n = 110). However, no significant difference was observed after 4-7 days (g = 0.09, 95% CI [-0.26; 0.44], p = 0.61) across five studies (n = 306). There was no significant difference in overall side effect rates between haloperidol and olanzapine (p = 0.29, 7 studies, n = 530), but haloperidol resulted in significantly more extrapyramidal side effects (p = 0.008). Sedation as an adverse effect did not differ significantly between the two drugs (p = 0.54, 4 studies, n = 284).

**Conclusions:**

Haloperidol may offer superior short-term efficacy in reducing delirium severity but is associated with a higher risk of extrapyramidal symptoms. No significant differences were found in long-term efficacy or sedation rates between olanzapine and haloperidol. These findings support the need for careful consideration of drug safety profiles in the treatment of delirium.

**Disclosure of Interest:**

None Declared

